# EV-derived miR-1246 promotes lymph node metastasis in esophageal squamous cell carcinoma through targeting the PPP2CB/NF-κB axis

**DOI:** 10.3389/fimmu.2026.1881297

**Published:** 2026-09-21

**Authors:** Yaojie Wang, Yuting Sun, Yuhui Fu, Zishuan Wei, Lianmei Zhao, Guoyou Zhang

**Affiliations:** 1 Department of Gastrocolorectal Surgery, Tongliao People’s Hospital, Tongliao, China; 2 Research Center, The Fourth Hospital of Hebei Medical University, Shijiazhuang, China; 3 Key Laboratory of Tumor Gene Diagnosis, Prevention and Therapy; Clinical Oncology Research Center, Shijiazhuang, Hebei, China

**Keywords:** esophageal squamous cell carcinoma, extracellular vesicles, lymph node metastasis, MiR-1246, NF-κB, PPP2CB

## Abstract

**Background:**

Lymph node metastasis (LNM) is a critical factor contributing to poor prognosis in esophageal squamous cell carcinoma (ESCC) patients. Consequently, elucidating its molecular mechanisms and identifying effective therapeutic targets have become a paramount challenge in clinical practice.

**Methods:**

Plasma samples were systematically collected from healthy individuals, ESCC patients without LNM, and those with LNM. Extracellular vesicles (EVs) were isolated from plasma by size-exclusion chromatography (SEC) and ultracentrifugation (UC) methods, and EV-derived miRNA expression profiles were detected and compared by sequencing. Differentially expressed miR-1246 was selected, and a diagnostic model based on plasma EV-miR-1246 was established to predict LNM. The biological functions of EV-miR-1246 and its target gene PPP2CB, which was identified by mass spectrometry combined with online target prediction methods, in the LNM of ESCC were further investigated using *in vitro* cellular assays and *in vivo* animal models. Rescue experiments and NF-κB agonist functional assays were performed to verify the downstream regulatory axis.

**Results:**

MiR-1246 levels were significantly elevated in plasma extracellular vesicles from ESCC patients with lymph node metastasis. The diagnostic model established based on vesicular miR-1246 showed favorable predictive efficacy for lymph node metastasis status in ESCC. *In vitro* functional experiments confirmed that vesicular miR-1246 derived from ESCC cells significantly enhanced the migration, invasion, and tube formation capacities of human lymphatic endothelial cells (HLECs). *In vivo* mouse experiments further confirmed that vesicular miR-1246 derived from ESCC cells significantly promoted lymph node metastasis of esophageal squamous cell carcinoma in mice. Mechanistically, miR-1246 targets and represses PPP2CB expression. Rescue experiments further confirmed that the miR-1246/PPP2CB axis activates NF-κB signaling, thereby promoting lymphatic endothelial activation and lymphangiogenesis.

**Conclusions:**

This study demonstrates that EV-derived miR-1246 acts as a promising predictive biomarker for lymph-node metastasis in ESCC. Mechanistically, EV-delivered miR-1246 targets PPP2CB to activate NF-κB signaling, thereby driving lymphatic endothelial activation and lymph node metastasis. These findings offer novel diagnostic biomarkers and therapeutic targets for ESCC patients with lymph node metastasis.

## 1 Introduction

Esophageal squamous cell carcinoma (ESCC) is a highly aggressive gastrointestinal malignancy with significant global incidence and mortality, particularly in East Asia (1). Early lymph node metastasis (LNM) contributes to its poor prognosis (2). Despite advances in surgical and comprehensive treatment strategies, the 5-year survival rate for ESCC patients with LNM remains substantially low (3). Therefore, elucidating the molecular mechanisms underlying LNM in ESCC and identifying effective biomarkers for early diagnosis and therapeutic targets represent critical challenges in current clinical practice.

Extracellular vesicles (EVs) are membrane-encapsulated nanoscale particles containing multiple cell-secreted biomolecules, including proteins, lipids, and nucleic acids. They have emerged as promising candidates for tumor diagnosis. Tumor-derived circulatory nucleic acid and protein showed obvious advantages in the early diagnosis, testing, and treatment of tumors (4, 5). Among them, miRNAs play an important role in the malignant process and drug resistance of tumors. In our previous study (6), we performed miRNA sequencing on plasma and plasma-derived EVs obtained from ESCC patients and healthy controls. From this dataset, we screened eight candidate miRNAs associated with LNM. Further qRT-PCR validation in a discovery cohort consisting of 20 healthy controls and 20 ESCC patients identified four miRNAs with stable dysregulated expression: plasma miR-636 and miR-7641, as well as EV-derived miR-1246 and miR-28-3p.

Further stratified validation was performed in an independent cohort of 140 ESCC patients with distinct LNM stages (N0 = 66, N1 = 42, N2–N3 = 32), followed by diagnostic-efficiency evaluation. Among the four candidates, EV-derived miR-1246 demonstrated the most stable expression signature and the best predictive performance for ESCC patients with LNM, and represented the major contributor to our four-miRNA diagnostic model (AUC = 0.804). Notably, EV-miR-1246 displays a stage-dependent biphasic expression profile: it is markedly elevated at the N1 early lymph-node-dissemination stage, while its expression declines in advanced N2–N3 subgroups, which is partially attributable to substantial inter-sample heterogeneity and progressive remodeling of circulating EV-miRNA profiles during late-stage tumor evolution (6). Although the other three candidates also possess biomarker potential, we prioritized EV-derived miR-1246 for subsequent mechanistic studies because of its superior diagnostic performance for detecting early-stage lymph-node metastasis.

Despite its encouraging diagnostic performance for ESCC patients with LNM, the exact biological functions and molecular mechanisms by which EV-derived miR-1246 facilitates ESCC lymph-node metastatic progression remain largely unclear. Accordingly, the present study was designed to dissect the regulatory role and underlying molecular mechanism of EV-derived miR-1246 in the progression of ESCC patients with LNM.

Herein, we first showed that Protein Phosphatase 2A (PP2A) is one of the targets of miR-1246 in ESCC. PP2A is a key tumor-suppressive phosphatase, and its functional inactivation is closely associated with poor prognosis in various cancers (7, 8). As the catalytic subunit of the PP2A holoenzyme, the expression level and enzymatic activity of PPP2CB directly determine the overall functional status of PP2A. More importantly, PP2A serves as a central signaling hub, and its functional impairment directly leads to the dysregulation of multiple oncogenic signaling pathways (9), among which the NF-κB signaling pathway is particularly critical. As a classic pro-oncogenic signaling pathway, the abnormal activation of NF-κB is one of the core molecular events in tumor lymph node metastasis. It can significantly promote the invasion, migration, and lymphangiogenesis of tumor cells by regulating the expression of downstream pro-metastatic genes (such as matrix metalloproteinases and vascular endothelial growth factor), thereby accelerating the process of lymph node metastasis. This mechanism has been widely confirmed in various malignant tumors, including esophageal squamous cell carcinoma (ESCC) (10, 11). From a clinical perspective, PP2A inactivation results in the loss of its “braking” effect on the AKT, ERK, and NF-κB signaling pathways, leading to the sustained activation of these pathways, which in turn synergistically promote cell survival, proliferation, and invasion (12). This not only enhances the invasiveness of tumor cells but also frequently confers resistance to conventional chemotherapeutic agents. Based on this mechanistic foundation, restoring or mimicking the tumor-suppressive function of PP2A has emerged as a promising novel therapeutic strategy.

In the present study, we confirmed that miR-1246 was significantly high in plasma-derived EVs from patients with LNM, and validated its diagnostic efficacy (AUC range: 0.7679–0.7941) in different cohorts. Furthermore, through *in vitro* and *in vivo* experiments, we further elucidated a molecular regulatory axis: ESCC cells deliver miR-1246 to human lymphatic endothelial cells (HLECs) *via* EVs, thereby directly targeting and suppressing the expression of PPP2CB to form the functional EV-miR-1246/PPP2CB axis. This axis induces the abnormal activation of the NF-κB signaling pathway by relieving the negative regulation of PPP2CB on NF-κB, ultimately promoting the migration, invasion, and lymphangiogenesis of HLECs, which provides an important molecular basis for LNM in ESCC. These findings provide novel insights and experimental evidence for the development of anti-metastatic strategies targeting the EV-miR-1246/PPP2CB axis to modulate the NF-κB signaling pathway.

## 2 Methods

### 2.1 Blood collection and plasma separation

Plasma samples from patients with esophageal squamous cell carcinoma and normal blood donors were both obtained from the Fourth Hospital of Hebei Medical University. This study was approved by the Ethics Committee of the Fourth Hospital of Hebei Medical University (Ethics Approval No. 2025KY258), and informed consent was obtained from all patients. Blood samples were collected *via* venipuncture into EDTA anticoagulant tubes, inverted and mixed 8–10 times, then centrifuged at 1500 × g for 20 minutes at 4 °C. The upper plasma layer was carefully aspirated into a new centrifuge tube and centrifuged again at 3000 × g for 15 minutes at 4 °C. The supernatant was carefully transferred to 2 mL cryopreservation tubes and either used immediately or stored in a -80 °C refrigerator.

### 2.2 Separation of plasma-derived EVs

Plasma samples (1 mL) were first filtered through a 0.22 μm filter (SLGPR33RB; Millipore). The supernatant was then fractionated using a Sepharose CL-2B size-exclusion chromatography (SEC) column (Echo9101A-5 mL; Echobiotech), which was eluted with PBS (P1020; Solarbio). Fractions of 500 μL were collected, and fractions 2–4, which typically contain EVs, were pooled. The pooled effluent was subjected to ultracentrifugation at 150,000 × g for 4 h. The resulting pellet was resuspended in PBS and subjected to a second round of ultracentrifugation under the same conditions for 2 h. Finally, the purified EVs were resuspended in PBS for downstream applications.

### 2.3 Characterization of plasma-derived EVs

We purified plasma EVs through sequential SEC and UC, and then validated the isolates by TEM, NanoFCM, and Western blotting in compliance with the MISEV 2018 guidelines (13).

#### 2.3.1 Transmission electron microscopy

Ten microliters of the plasma EV suspension were dropped onto a carbon-coated copper grid and allowed to stand at room temperature for 10 minutes. The copper grid was rinsed with sterile distilled water, and excess liquid was blotted off with filter paper. The grid was then placed in a uranyl acetate solution (pH = 7) and left to stand at room temperature for 1 minute. Excess liquid on the grid was removed with filter paper, followed by air-drying at room temperature for 2 minutes. The morphology of EVs was observed and photographed using TEM (Hitachi HT7800; Hitachi Limited).

#### 2.3.2 Nano-flow cytometry

The parameters of the nano-flow cytometer were calibrated using the concentration and particle size of standard samples before use. The size and concentration of EV samples were measured repeatedly with the nano-flow cytometer (U30; NanoFCM), and 1-minute results were collected. The particle size and concentration of EV samples were analyzed based on the standard samples, and the particle size distribution map of EVs was obtained.

#### 2.3.3 Protein extraction and western blotting analysis

RIPA buffer containing PMSF (R0010; Solarbio) was added to the EVs, followed by vortexing to mix well, and the mixture was left standing on ice for 20–30 minutes. After centrifugation at 12,000 rpm for 30 minutes at 4 °C, the EV protein supernatant was aspirated into a new EP tube and stored at -80 °C. The concentration of EV proteins was determined using the BCA method, and the concentration of the EV protein samples was calculated accordingly. The volume of the protein samples was accurately measured with a pipette, and 5 × loading buffer was added based on the sample volume. After thorough mixing, the samples were placed in a 100 °C dry bath and boiled for 5 minutes to ensure complete denaturation of the proteins. After removal, the samples were cooled to room temperature and stored at -20 °C. Total protein (60 μg) was separated using a 12% sodium dodecyl sulfate-polyacrylamide gel (SDS-PAGE), and then transferred onto polyvinylidene fluoride (PVDF) membranes (ISEQ00010; Millipore). Next, the membranes were blocked with 5% nonfat milk powder prepared in Tris Buffered Saline containing 0.1% Tween-20 (TBST). After blocking, the membranes were incubated overnight with specific primary antibodies targeting CD9 (1: 200, sc-13118; Santa Cruz), TSG101 (1: 200, sc-7964; Santa Cruz), HSP90 (1: 2000, 13171-1-AP; Proteintech), and Calnexin (1: 200, sc-23954; Santa Cruz). Following three washes with TBST, the membranes were further incubated for 1 h with fluorochrome-conjugated secondary antibodies (anti-rabbit/anti-mouse IgG). Finally, the membranes were visualized and imaged using the Odyssey Infrared Imaging System (9120; LI-COR).

### 2.4 RNA extraction and sequencing

miRNA was extracted and purified from plasma and plasma EVs using the miRNeasy^®^ Mini Kit (217004; Qiagen). Library construction was performed with the QIAseq miRNA Library Kit (331505; Qiagen); index codes were added to the attribute sequences of each sample. The quality of the libraries was assessed using an Agilent Bioanalyzer 2100 and qRT-PCR. Clustering was carried out on the cBot Cluster Generation System with the TruSeq PE Cluster Kit v3-cBot-HS. After cluster generation, the libraries were sequenced on the Illumina HiSeq platform, generating 150 bp reads.

### 2.5 Reverse-transcription and quantitative real-time PCR

Reverse transcription of miRNAs into cDNA was conducted using the Primer-Script™ RT reagent kit (RR037A; TAKARA). For amplification of the target gene, qRT-PCR was performed with the TaqMan™ probe kit (RR390A; TAKARA), and U6 was used as the internal reference gene for normalizing miRNA expression levels. For the newly expanded validation cohort, synthetic cel-miR-39-3p spike-in control (sequence 5’-UCACCGGGUGUAAAUCAGCUUUG-3’; YE0223920, RiboBio) was spiked into each sample after RNA extraction for exogenous normalization to correct technical variations. Both cel-miR-39-3p and U6 were applied for normalization of miR-1246 expression.

mRNA was reverse-transcribed into cDNA using the Promega reverse transcription kit (A5000; Promega), and the target genes were amplified by qPCR with the GoTaq^®^ qPCR Master Mix kit (A6001; Promega). GAPDH served as the internal reference gene for normalizing mRNA expression levels. Primers were sourced from Sangon Biotech Co., Ltd., and their details are provided in **Table 1**.

**Table 1:** Sequences of primer and probe (5’-3’).

ID	Forward primers	Reverse primers	Probes	Stem-loop RT primers
miR-1246	ACGGAGCGAATGGATTTTTGG	GTGCAGGGTCCGAGGT	CAGAGCCACCTGGGCAATTT	CAGTGCAGGGTCCGAGGTCAGAGCCACCTGGGCAATTTTTTTTTTTCCTGC
U6	CTCGCTTCGGCAGCACA	GTGCAGGGTCCGAGGT	AGAAGATTAGCATGGCCCCTGCGCA	GTGCAGGGTCCGAGGT
PPP2CB	ATTCAGTGGAGACTGTGACTCT	GGCTTTCGTGATTTCCTCTCAAT		
GAPDH	CGCTGAGTACGTCGTGGAGTC	GCTGATGATCTTGAGGCTGTTGTC		
cel-miR-39-3p	GCGTCACCGGGTGTAAATC	AGTGCAGGGTCCGAGGTATT		GTCGTATCCAGTGCAGGGTCCGAGGTATTCGCACTGGATACGACCAAGCT

### 2.6 Cell lines and cell culture

The ESCC cell line TE-1 was acquired from the Shanghai Institute of Cell Biology, Chinese Academy of Sciences (Shanghai, China). The KYSE-150 ESCC cell line was kindly provided by the laboratory of Qimin Zhan at the Cancer Hospital, Chinese Academy of Medical Sciences (Beijing, China). All ESCC cells were maintained in Roswell Park Memorial Institute (RPMI)-1640 medium (C11875500BT; GIBCO). This medium was supplemented with 10% heat-inactivated fetal bovine serum (FBS) (04-001-1ACS; Biological Industries), and 1% penicillin-streptomycin (PS) (P1400; Solarbio). The HLECs were purchased from the BeNa Culture Collection (BNCC, China). These HLECs were cultured in Endothelial Cell Medium (1001; ScienCell), which was supplemented with 10% heat-inactivated FBS, 1% PS, and 1% endothelial cell growth supplement (1052; ScienCell). All cell lines were incubated at 37 °C in a humidified incubator with a 5% CO_2_ atmosphere.

### 2.7 Collection and isolation of EVs from cell supernatants

Cell supernatants were collected and centrifuged at 3000 × g for 15 minutes to remove cell debris. The supernatants were then added to 100 kDa Amicon Ultra-15 ultrafiltration tubes and centrifuged at 3500 × g for 10 minutes at room temperature until all collected supernatants were concentrated to less than 50 mL. Subsequently, EVs from the cell supernatants were isolated using the SEC column as described previously.

### 2.8 Uptake of EVs

The uptake of EVs derived from KYSE-150 and TE-1 cells by HLECs was detected using the PKH67 EV Labeling Kit (MINI67-1KT; Thermo). Briefly, 50 μg of EVs was mixed with 20 μL of PKH67 dye and stained at room temperature for 3 minutes. EV-free medium was added to terminate the staining, and the EVs were collected after being washed twice by ultrafiltration tube filtration. The stained EVs were added to confocal dishes and co-incubated with HLECs for 3 h. After washing three times with PBS, the cells were fixed with 4% paraformaldehyde for 20 minutes, followed by one PBS wash. DAPI was added for 10 minutes to stain cell nuclei, and after PBS washing, the uptake of EVs by HLECs was observed under a laser scanning confocal microscope (LSM510; ZEISS).

### 2.9 Cell transfection

#### 2.9.1 Transfection with siRNA

miRNA inhibitors, mimics, negative controls, and PPP2CB siRNA were all purchased from GenePharma. KYSE-150, TE-1, and HLECs in exponential growth phase were seeded into 6-well plates before transfection. When the cell confluency reached 70%-80%, transfection was performed according to the instructions of the Lipofectamine 2000 transfection reagent (11668019; Thermo Fisher).

#### 2.9.2 Transfection with plasmid

The overexpression plasmid of PPP2CB was purchased from Shanghai GeneChem Co., Ltd. HLEC cells in exponential growth phase were seeded into 6-well plates before transfection. When the cell confluency reached 70%-80%, plasmid transfection was performed according to the instructions of the Neofect transfection reagent.

#### 2.9.3 Transfection with Lentivirus

Lentiviruses were constructed by Shanghai GeneChem Co., Ltd. KYSE-150 and TE-1 cells in exponential growth phase were seeded at a density of 5 × 10^4^ cells/mL into 6-well plates before lentiviral infection, and cultured overnight in a constant-temperature incubator at 37 °C with 5% CO_2_. At the time of transfection, the original medium in the 6-well plates was discarded and replaced with 1 mL of fresh medium. The corresponding volume of lentivirus was calculated using the formula: Viral Volume = (MOI × Number of Cells)/Viral Titer, and polybrene was added simultaneously. At 24 h post-lentiviral infection, the medium was replaced with fresh complete RPMI-1640 medium containing 10% FBS, and the cells were further cultured in the constant-temperature incubator at 37 °C with 5% CO_2_. At 72 h post-lentiviral infection, the infection efficiency of the cells was observed under a fluorescence microscope. Successful lentiviral infection was confirmed when the cell infection efficiency exceeded 80%.

### 2.10 Wound healing assay

In brief, 5 × 10^5^ cells were plated into six-well plates and cultured in medium supplemented with 10% FBS. Next, the cells were transfected with siRNA, plasmid, or lentiviruses, respectively. Once the cells had grown to 80% confluence, a sterile 200 μL pipette tip was used to scratch a uniform, straight line through the cell monolayer in each well, generating a standardized wound gap. Thereafter, the cells were incubated in fresh serum-free medium. An inverted microscope (10 ×) was utilized to capture images of the wound closure at 0 and 24 h. Finally, the width of the remaining wound gap was quantified using Image-Pro Plus 6.0 software.

### 2.11 Transwell migration and invasion assay

The cells transfected with siRNA, plasmid, or lentiviruses were plated into the upper chambers of 24-well transwell inserts (8 μm pore size; Corning Costar) in 200 μL of serum-free RPMI-1640 medium. For the lower chambers, 600 μL of medium containing 20% FBS was added to act as a chemoattractant. Following 15–20 h of incubation, cells on the upper surface of the inserts were wiped away using cotton-tipped swabs. In contrast, cells that had moved to the lower surface were fixed with 4% formaldehyde and stained with 0.05% crystal violet for 10 minutes. The procedure for the cell invasion assay was comparable to that of the cell migration assay, with one distinction: transwell units were precoated overnight with 200 μg/mL Matrigel (356234; Becton) before the assay. Cells attached to the lower surface of the insert membrane were identified as migrated or invaded cells. These cells were imaged and counted under a microscope at 200 × magnification.

### 2.12 Lymphangiogenesis assay

Prior to the experiment, matrigel was taken out from the -20 °C refrigerator and placed in a 4 °C refrigerator for overnight thawing until it naturally liquefied. The 24-well plates and pipette tips used in the experiment were pre-cooled in a -20 °C refrigerator for 2 h before operation, and a low-temperature environment was maintained throughout the operation. 30 μL of pre-cooled Matrigel was slowly added to the pre-cooled 24-well plates to avoid generating air bubbles. The 24-well plates were then placed in a 37 °C constant-temperature incubator and left to stand for 2 h to allow the Matrigel to fully solidify. HLECs from each treatment group were collected, digested, centrifuged, and resuspended. The cell density was adjusted to 1 × 10^5^ cells per well, and the cell suspension of each group was slowly added dropwise into the 24-well plates, with care taken to avoid air bubbles. The cells were cultured continuously in a 37 °C, 5% CO_2_ constant-temperature incubator. Images were taken under an inverted phase-contrast microscope within 12 h after seeding, and the number of lymphatic tubules in high-power fields was calculated using ImagePro Plus software.

### 2.13 Proteomic analysis

The peptides (1 µg per sample) were loaded into a nanoflow HPLC Easy-nLC1200 system, with a 90-minute LC gradient applied at a flow rate of 300 nL/min. Buffer A was composed of water containing 0.1% (v/v) formic acid (FA), and Buffer B was composed of 80% acetonitrile (ACN) containing 0.1% (v/v) FA. The gradient was set as follows: 2%-8% Buffer B for 1 min; 8%-28% Buffer B for 60 min; 28%-37% Buffer B for 14 min; 37%-100% Buffer B for 5 min; and 100% Buffer B for 10 min. Proteomic analysis was conducted on a Q Exactive HF mass spectrometer. The spray voltage was set to 2.3 kV in positive ion mode, and the ion transfer tube temperature was set to 320 °C. Data acquisition was performed using Xcalibur software. All raw data were processed in Proteome Discoverer 2.2 using either the ion-based label-free quantification method or the basic protein identification method described in the literature. Peptides were identified via Sequest HT, and all data were searched against the UniProt human reference database. Multiple testing correction was implemented by calculating the false discovery rate (FDR). An FDR of 1% was applied at both the peptide-spectrum match and protein group levels. The quantitative ratio of each peptide was determined through pairwise analysis, and the average values were then calculated at the peptide group and protein levels. Significance was determined by analysis of variance (ANOVA) based on peptide background and protein levels. Differentially expressed proteins were selected under the criteria of *FC > 1.5* or *FC < 0.5*. Proteomic analysis results were visualized using HemI software.

### 2.14 Dual-luciferase reporter gene assay

The binding site of miR-1246 and PPP2CB 3’UTR was searched using the online miRNA target gene prediction website miRDB. Based on this, wild-type (WT) and mutant (MT) plasmids of PPP2CB were designed:

The sequence of WT plasmid is as follows: TTTGCGCTTATTTGGAAATTTTAGTTATAGTGTTTAACTGGCATGGATTAATAGAGTTGGAGTTTTATTTTTAAGAAAAATTCACAAGCTAACTTCCACTAATCCATTATCCTTTATTTTATTGAAATGTATAATTAACTTAACTGAAGAAAAGGTTCTTCTTGGGAGTATGTTGTCATAACATTTAAAGAGATTTCCCTTCATTTA.

The sequence of MT plasmid is as follows: TTTGCGCTTATTTGGAAATTTTAGTTATAGTGTTTAACTGGCATGGATTAATAGAGTTGGAGTTTTATTTTTAAGAAAAATTCACAAGCTAACTTCCACTCCGAACGTATCCTTTATTTTATTGAAATGTATAATTAACTTAACTGAAGAAAAGGTTCTTCTTGGGAGTATGTTGTCATAACATTTAAAGAGATTTCCCTTCATTTA.

Plasmid transfection was performed according to the instructions of Neofect transfection reagent. Dual-luciferase activity was detected following the protocol of the Dual-Luciferase Reporter Gene Kit (E1910, Promega).

### 2.15 PMA pretreatment

HLECs were pretreated with PMA (HY-18739; MCE) for 24 h. HLECs treated with an equivalent concentration of DMSO (RS1044; Report BIOTECH) served as controls. After pretreatment, cells were harvested and subjected to tube-formation assay according to the standard procedure described in Section 2.12. Cell lysates were also prepared for western blot assay performed following the standard protocol described in Section 2.3.3.

### 2.16 Development of the popliteal lymph node metastasis model in nude mice

KYSE-150 cells were collected and resuspended in PBS, and the cell density was adjusted to 2.5 × 10^7^ cells/mL. The cell suspension was transferred to a sterile Eppendorf tube, placed on ice to reduce cell metabolism, and inoculated within half an hour. The footpads and legs of nude mice were thoroughly disinfected with iodophor cotton balls/alcohol. The cell suspension was gently pipetted to mix well to prevent cell clumping. Tumor cells were slowly injected into the footpads of nude mice, and attention was paid to pressing the injection site with a sterile cotton swab for 2 minutes when withdrawing the needle to avoid tumor formation failure caused by cell leakage. When visible tumors formed at the footpads (approximately 3 weeks), multi-point injections of EVs derived from each group of esophageal squamous cell carcinoma cells resuspended in sterile normal saline were administered intratumorally, at 50 μg per injection per mouse, twice a week for approximately 2 weeks. When the experimental requirements were met, the nude mice were sacrificed by cervical dislocation. The primary footpad tumors and popliteal lymph node tumors were collected. The long and short diameters of the footpad tumors and lymph node tumors were accurately measured using a vernier caliper, and their weights were measured using an electronic balance. The footpad tumors and lymph node tumors were rinsed with normal saline, fixed in 10% paraformaldehyde, and stored at 4 °C.

### 2.17 Immunohistochemical staining assay

All tissue specimens were embedded in paraffin, and 4 μm-thick pathological sections were prepared. Immunohistochemical staining was performed using the SP method, followed by observation and analysis under an upright microscope. In 5 randomly selected high-power fields (× 400), the percentage of positive cells was counted separately: ≤ 25% was scored as 0, 26–50% as 1, 51–75% as 2, and > 75% as 3. The staining intensity was then scored: no coloration as 0, light tan as 1, tan as 2, and dark brown as 3. The sum of the two scores was used for further interpretation: 0 as (–), 1–2 as (+), 3–4 as (++), and 5–6 as (+++). In this experiment, both (++) and (+++) were defined as positive expression, while (–) and (+) were defined as negative expression. All tissue sections were independently reviewed and interpreted by two pathologists.

### 2.18 Hematoxylin-eosin staining

Paraffin-embedded tissue sections (4 μm) were deparaffinized and rehydrated. Sections were stained with hematoxylin, followed by eosin counterstaining. After gradient dehydration and clearing, sections were mounted using neutral balsam. Tissue morphology was examined under an upright light microscope, and representative histological images were acquired for morphological assessment.

### 2.19 Statistical analysis

All statistical analyses were conducted using GraphPad Prism (version 8.3.0) and SPSS (IBM, version 22.0). ANOVA was employed to compare the differentially expressed miRNAs in plasma-derived EVs of ESCC patients with different LNM stages, with a p-value < 0.05 regarded as statistically significant. Heatmaps were constructed using the heatmap package (version 3.5.1). The student’s t-test was utilized to compare gene expression levels, and miR-1246 expression data was log-transformed prior to the t-test. For qRT-PCR detection, GAPDH and U6 were utilized as endogenous reference genes for cellular samples. For EV-derived miRNA quantification, data normalization was performed using both exogenous cel-miR-39-3p spike-in control and U6. MiR-1246 was normalized separately against each reference. Samples with cel-miR-39-3p Ct values deviating > ± 2 cycles from the cohort median were excluded due to unsatisfactory RNA recovery. Relative expression was calculated with the 2^-ΔCt^ method. ROC curves were generated based on miR-1246 expression values, and the AUC with a 95% confidence interval was computed for each curve. GO and KEGG functional enrichment analyses were conducted on differentially expressed genes as well as the 100 potential target genes of miR-1246 identified *via* HPLC-MS using the DAVID online platform (https://davidbioinformatics.nih.gov/). Pearson’s correlation analysis was applied to examine the correlation between intracellular miR-1246 levels and miR-1246 levels derived from cellular EVs. Gene expression data and corresponding clinicopathological information of ESCC were downloaded from the TCGA-ESCA database (https://portal.gdc.cancer.gov/). We analyzed the expression of PPP2CB in ESCC samples with lymph node metastasis versus those without, along with its correlation with survival outcomes. The target genes of miR-1246 were predicted using the online miRNA target gene prediction websites miRDB and TargetScan, respectively. The target genes obtained from these two prediction websites and those identified by mass spectrometry were intersected using the Venn diagram online website (http://jvenn.toulouse.inra.fr/app/example.html).

## 3 Results

### 3.1 Plasma EV-derived miR-1246 serves as a biomarker for diagnosing lymph node metastasis in esophageal squamous cell carcinoma

To confirm that the isolated particles from plasma were indeed EVs, we characterized them using transmission electron microscopy (TEM), nanoparticle flow cytometry (NanoFCM), and western blotting. The results demonstrated that the isolated particles exhibited a typical cup-shaped morphology with a double-membrane structure (**Figure 1A**), a particle size distribution ranging from 40 to 180 nm (**Figure 1B**), and positive expression for EV-specific markers (CD9, HSP90, TSG101) while being negative for the non-EV marker Calnexin (**Figure 1C**). These findings collectively confirm the successful isolation of EVs from plasma. Subsequently, to identify differentially expressed plasma EV-derived miRNAs with predictive value for lymph node metastasis in ESCC, we performed next-generation sequencing on plasma EVs from 10 healthy donors and 22 ESCC patients with varying LNM statuses (N0 = 13, N1 = 5, N2 = 3, N3 = 1). Sequencing analysis revealed 109 differentially expressed miRNAs (6). Among these, miR-1246 was significantly upregulated in the plasma EVs of ESCC patients with LNM compared to both healthy donors and ESCC patients without LNM (*P = 0.042*, **Figure 1D**). We further validated the expression level of miR-1246 in a training cohort comprising 90 ESCC plasma EV samples (47 with LNM *vs*. 43 without LNM) and a validation cohort of 58 ESCC plasma EV samples (34 with LNM *vs*. 24 without LNM). Consistent with the sequencing data, miR-1246 expression was significantly higher in the plasma EVs of ESCC patients with LNM than in those without LNM (**Figures 1E, F**). In the training cohort, qRT-PCR data of miR-1246 were normalized to the endogenous reference U6. In the test cohort, we adopted two parallel normalization strategies: endogenous U6 and the exogenous spike-in control cel-miR-39-3p. This experimental design enabled us to objectively compare the quantification performance of spike-in (cel-miR-39) and the conventional endogenous reference U6 and further verify the expression of miR-1246. Intriguingly, miR-1246 exhibited a significant up-regulation trend in patients with LNM regardless of whether exogenous or endogenous normalization was applied, further confirming the reliability of our qRT-PCR results. Subsequently, based on the above qRT-PCR results, we constructed diagnostic models for ESCC with LNM using the expression levels of plasma EV-derived miR-1246. As shown in **Figure 1E**, the model based on plasma EV-derived miR-1246 achieved areas under the curve (AUC) of 0.7679 in the U6-normalized training cohort. In the test cohort, comparable AUC values were achieved: 0.7941 for U6-normalized miR-1246 and 0.7917 for cel-miR-39-3p-normalized miR-1246 (**Figure 1F**). These results indicate that miR-1246 is differentially expressed in the plasma EVs of ESCC patients with and without lymph node metastasis, suggesting its potential utility as a diagnostic biomarker for identifying LNM in ESCC.

**Figure 1:**
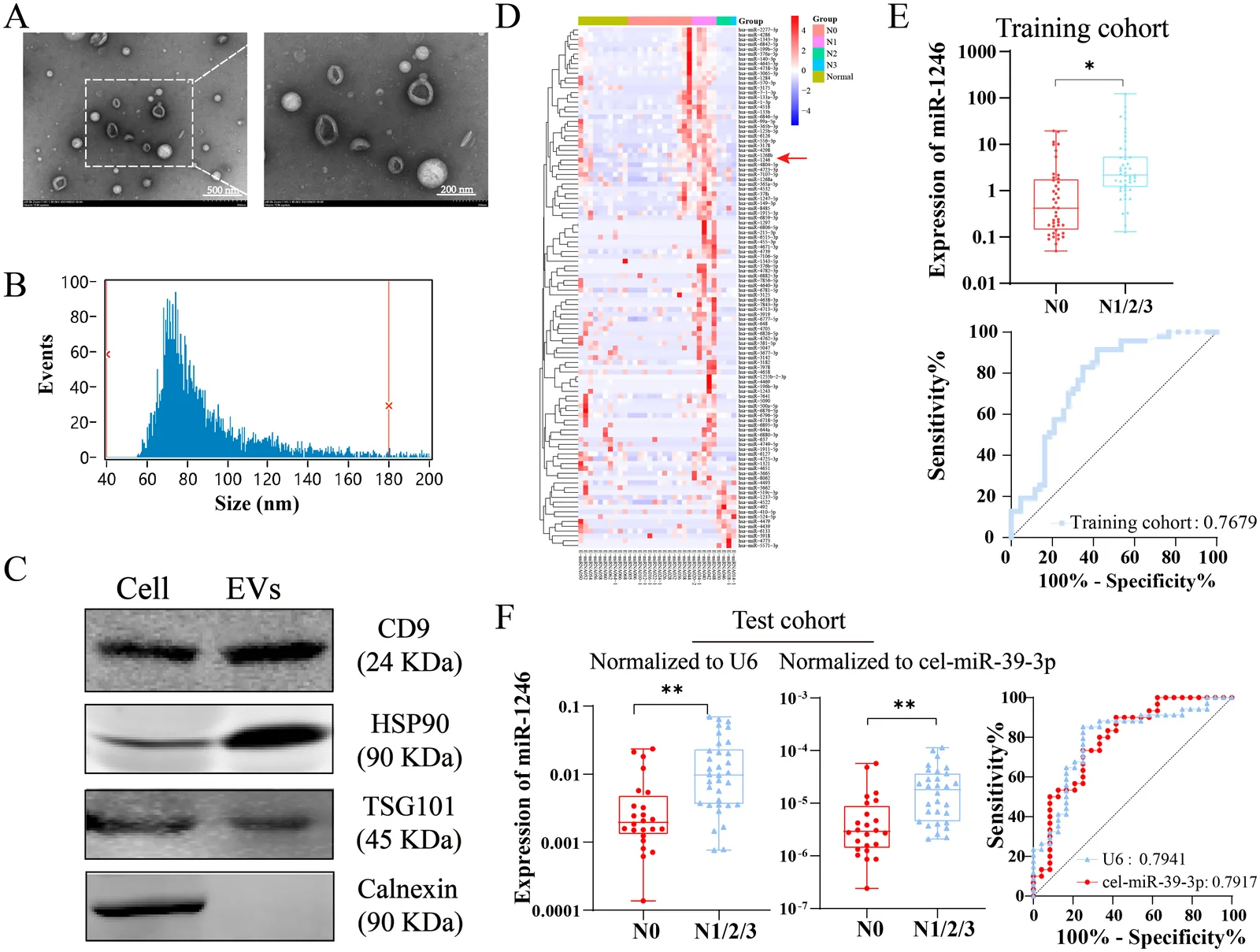
Plasma EV-derived miR-1246 serves as a biomarker for diagnosing lymph node metastasis in esophageal squamous cell carcinoma. **(A)** Images of transmission electron microscopy showing that EVs are bowl-shaped. Scale, 200 nm and 500 nm. **(B)** Nano-FCM analysis showing the size distribution of EVs; the mean size was 87.67 nm. **(C)** Western blot analysis showing the expression of CD9, HSP90, and TSG101, and the absence of Calnexin. **(D)** Heatmap of differentially expressed miRNAs (Normal *vs.* N0 *vs.* N1 *vs.* N2 *vs.* N3) in plasma-derived EVs. **(E)** Plasma EV-miR-1246 expression and the corresponding ROC curve in the training cohort of ESCC patients without lymph node metastasis (N0) and with lymph node metastasis (N1/2/3). *P < 0.05. **(F)** Plasma EV-miR-1246 expression normalized to U6 and cel-miR-39-3p, as well as their corresponding ROC curves, in the test cohort. **P < 0.01.

### 3.2 EV-derived miR-1246 from esophageal squamous cell carcinoma promotes lymphangiogenesis *in vitro*

EVs secreted by tumor cells can be taken up not only by other tumor cells but also by various cells within the tumor microenvironment, such as immune cells, vascular endothelial cells, and lymphatic endothelial cells (LECs) (14–16). This process regulates key biological functions in recipient cells, including immune responses, angiogenesis and lymphangiogenesis, thereby fostering a microenvironment conducive to tumor growth. Research has established that lymphangiogenesis is crucial for lymph node metastasis, as newly formed lymphatic vessels possess loose intercellular junctions and high permeability, facilitating tumor cell intravasation. Consequently, higher lymphatic vessel density is associated with the LNM.

Given our previous finding that plasma EV-derived miR-1246 levels correlate significantly with LNM status, we sought to explore its functional role and mechanism in LNM of ESCC. We co-cultured EVs secreted by KYSE-150 and TE-1 ESCC cells with HLECs. Confocal laser scanning microscopy confirmed the uptake of these tumor-derived EVs by HLECs (**Figure 2A**).

**Figure 2:**
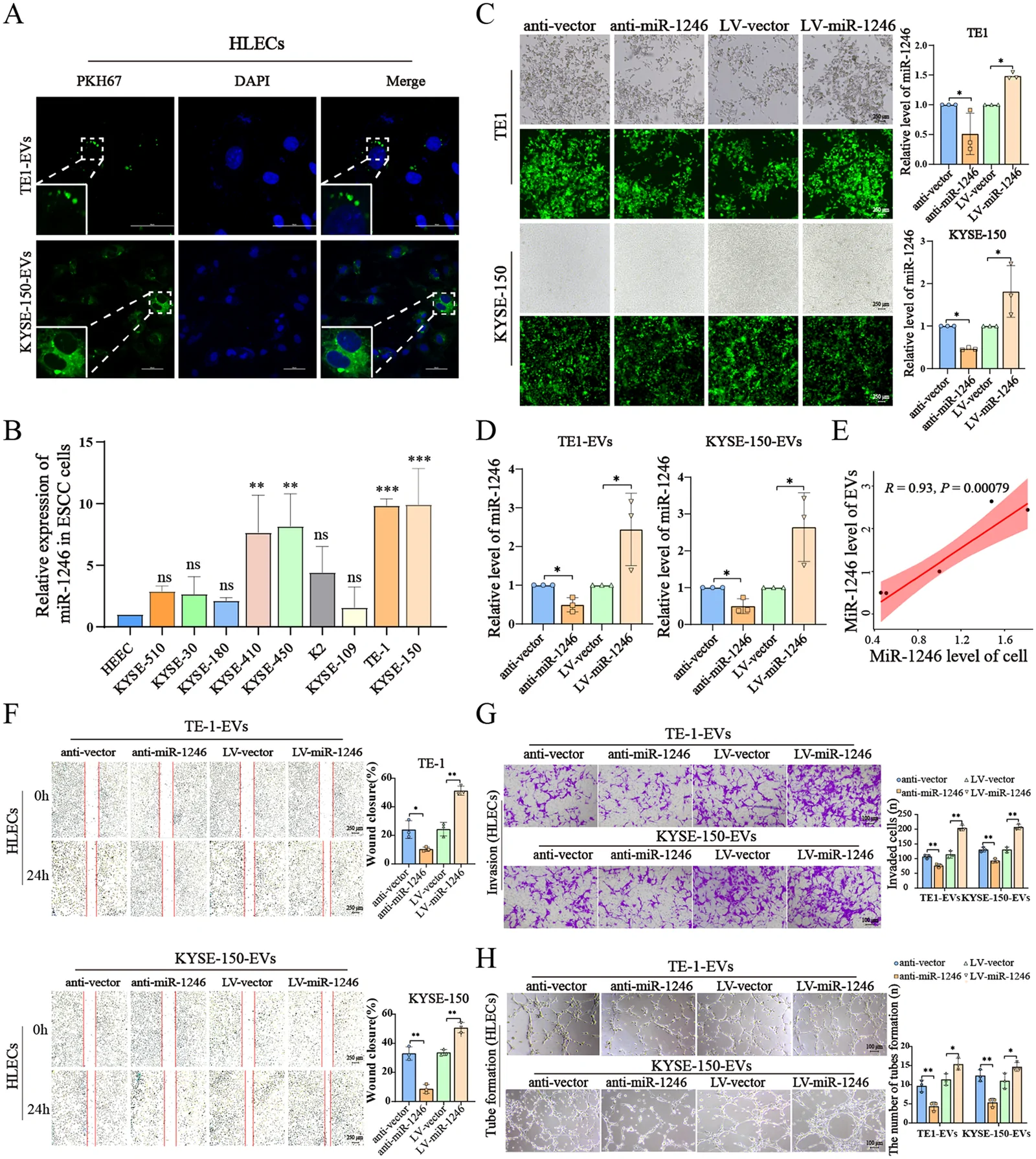
EV-derived miR-1246 from esophageal squamous cell carcinoma promotes lymphangiogenesis *in vitro*. **(A)** Detected the uptake of ESCC cell-derived EVs by HLECs using EVs uptake assay. **(B)** Detection of miR-1246 expression in each cell line using qRT-PCR. **(C)** Transfection efficiency of lentivirus overexpressing or knocking down miR-1246 in KYSE-150 and TE-1 cells observed under fluorescence microscopy, and the expression was detected by qRT-PCR. **(D)** Expression levels of miR-1246 in cell-derived EVs of each transfection group were detected by qRT-PCR. **(E)** Correlation of miR-1246 expression levels in each transfected group of cell lines with those in their EVs using Pearson correlation analysis. **(F–H)** Effect of miR-1246 derived from EVs on migration, invasion, and lymphatic vessel formation of HLECs detected by wound healing assays **(F)**, Matrigel invasion assays **(G)**, and lymphatic vessel formation assays **(H)**.

To investigate the impact of EV-derived miR-1246 on HLECs function, we first assessed miR-1246 expression levels. qRT-PCR analysis revealed significantly upregulated miR-1246 expression in several ESCC cell lines (TE-1, KYSE-410, KYSE-450 and KYSE-150) compared to the normal esophageal epithelial cell line HEEC (*P < 0.05*) (**Figure 2B**). KYSE-150 and TE-1 cells, which exhibited the highest expression, were selected for generating stable cell lines with either overexpression or knockdown of miR-1246. Successful transduction was confirmed by the presence of green fluorescent protein, and significant alterations in miR-1246 expression levels in the modified cells (LV-miR-1246 *vs.* LV-vector; anti-miR-1246 *vs.* anti-vector) were verified by qRT-PCR (*P < 0.05*) (**Figure 2C**).

We next measured miR-1246 levels in EVs isolated from these engineered cells. EVs from overexpression groups (KYSE-150/TE-1-_LV-miR-1246_-EVs) contained significantly higher levels of miR-1246 than control EVs (KYSE-150/TE-1-_LV-vector_-EVs). Conversely, EVs from knockdown groups (KYSE-150/TE-1-_anti-miR-1246_-EVs) showed significantly lower miR-1246 levels compared to their respective controls (KYSE-150/TE-1-_anti-vector_-EVs) (*P < 0.05*) (**Figure 2D**). Additionally, we performed Pearson correlation analysis between the expression levels of miR-1246 in each transfected cell line and those in their derived EVs. The results showed a significant positive correlation between the expression levels of miR-1246 in each transfected cell line and those in their EVs (*R = 0.93, P = 0.00079*, **Figure 2E**), supporting the role of EVs as carriers for miR-1246 delivery.

Subsequent functional assays, including scratch wound healing, matrigel invasion, and lymphatic tube formation, demonstrated that co-culture of HLECs with EVs derived from miR-1246-overexpressing KYSE-150/TE-1 cells for 24 h significantly enhanced their migratory, invasive, and lymphangiogenic capacities compared to the control groups. Conversely, HLECs co-cultured with EVs from miR-1246-knockdown cells exhibited a significant impairment in these capabilities (*P < 0.05*; **Figures 2F–H**). In summary, these results indicate that ESCC cell-derived EVs carrying elevated levels of miR-1246 promote the migratory, invasive, and lymphangiogenic activities of HLECs *in vitro*, while EVs with reduced miR-1246 exert inhibitory effects.

### 3.3 PPP2CB is the target gene of miR-1246

To elucidate the mechanism by which miR-1246 mediates LNM in ESCC, we overexpressed miR-1246 in HLECs and screened for differentially expressed proteins using HPLC-MS. Applying criteria of fold change (FC) *< 0.5* and *P < 0.05*, we identified 100 potential target genes of miR-1246 (**Figure 3A**).

**Figure 3:**
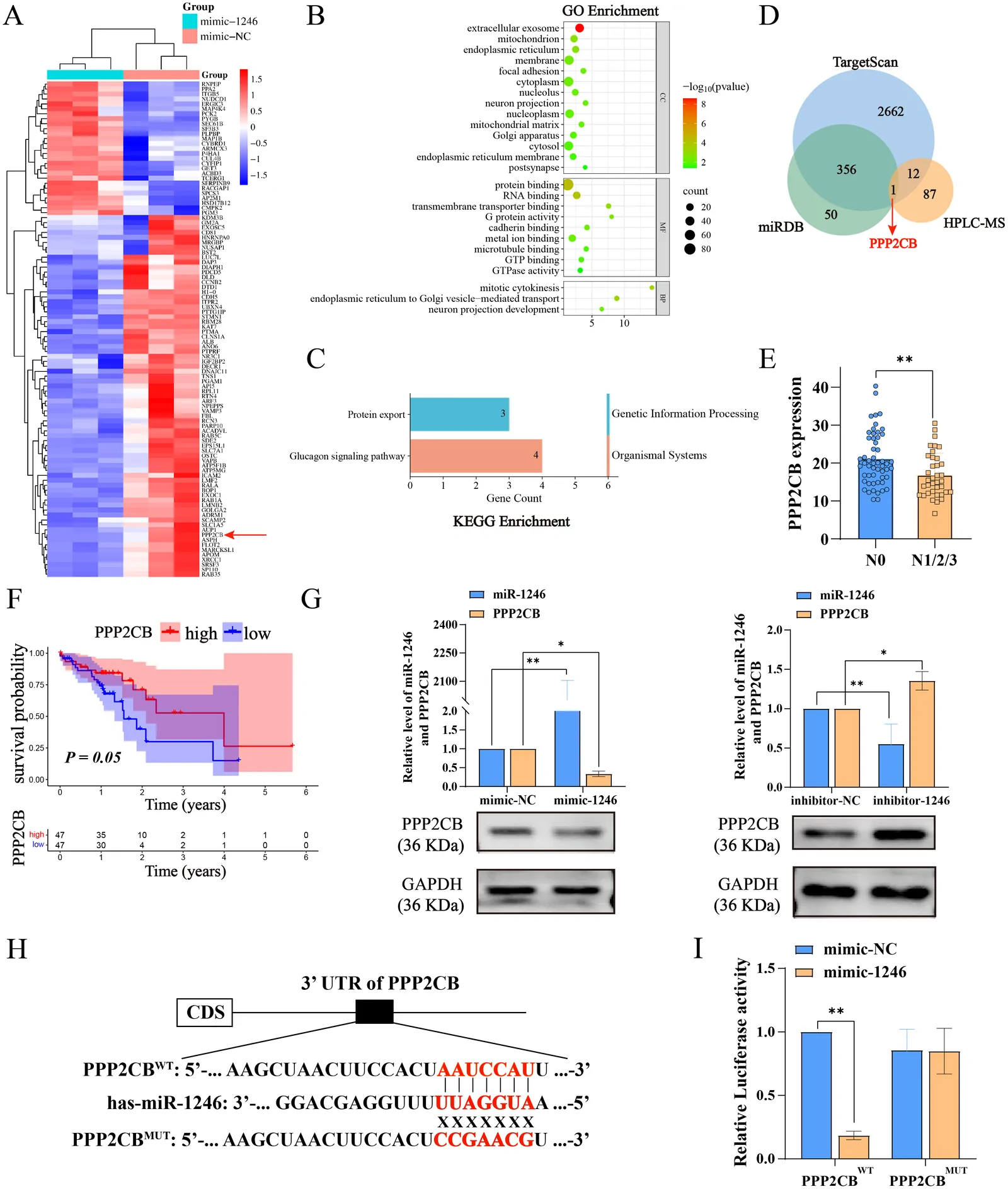
PPP2CB is the target gene of miR-1246. **(A)** Detection of miR-1246 target genes by HPLC-MS. **(B)** Gene Ontology (GO) enrichment analysis of the 100 candidate target genes of miR-1246. **(C)** KEGG pathway enrichment analysis of miR-1246 candidate target genes. **(D)** The Venn diagram of miR-1246 target genes detected by HPLC-MS and predicted by miRDB and TargetScan database. **(E)** The expression of PPP2CB in lymph node metastasis and non-metastasis samples of ESCC was analyzed by the TCGA database. **(F)** The expression of PPP2CB on the survival of ESCC patients was analyzed by TCGA database. **(G)** Effect of overexpression or knockdown of miR-1246 on the level of PPP2CB in HLECs by qRT-PCR and Western blotting. **(H)** Schematic graph of wild-type and mutant putative binding sites of miR-1246 targeting the 3’-UTR of PPP2CB. **(I)** Relative luciferase activity of HEK293T cells after co-transfection with wild-type or mutant PPP2CB 3’-UTR reporter genes and miR-1246-mimic.

To gain insight into the biological functions and pathways associated with 100 potential target genes, we performed Gene Ontology (GO) and Kyoto Encyclopedia of Genes and Genomes (KEGG) enrichment analyses. GO enrichment analysis of cellular component (CC) terms showed that the target genes were predominantly enriched in extracellular exosomes. Other top-ranked enriched compartments included the mitochondrion, endoplasmic reticulum, cell membrane, focal adhesion, and cytoplasm (**Figure 3B**). In the molecular function (MF) category, the genes were predominantly associated with protein binding and RNA binding, with additional enrichment in transmembrane transporter binding, G protein activity, cadherin binding, metal ion binding, microtubule binding, GTP binding, and GTPase activity (**Figure 3B**). In the biological process (BP) category, the enriched terms included mitotic cytokinesis, endoplasmic reticulum to Golgi vesicle-mediated transport, and neuron projection development (**Figure 3B**). KEGG pathway analysis identified two significantly enriched pathways: protein export and glucagon signaling pathway (**Figure 3C**).

Concurrently, we employed the online miRNA target prediction databases miRDB and TargetScan, which predicted 407 and 3031 putative targets of miR-1246, respectively. A Venn diagram analysis revealed a single overlapping gene, PPP2CB, from these computational predictions (**Figure 3D**). Notably, PPP2CB was also the most significantly downregulated gene identified by HPLC-MS (*FC = 0.028, P = 0.015*).

Previous studies indicate that PPP2CB is an isoform of the catalytic subunit of PP2A. PP2A is a major serine/threonine phosphatase that counteracts kinase activity through dephosphorylation, thereby regulating numerous critical signaling pathways (17). In cancer, the PP2A complex is generally considered a tumor suppressor, and its dysfunction can promote tumorigenesis, progression, invasion, and metastasis (18).

We next analyzed gene expression data and corresponding clinicopathological information from the TCGA-ESCA database. This analysis showed that PPP2CB mRNA expression was significantly lower in ESCC samples with LNM compared to those without metastasis (*P < 0.01*) (**Figure 3E**), suggesting a significant negative correlation between PPP2CB and LNM. Furthermore, survival analysis indicated that ESCC patients with high PPP2CB expression had a significantly longer overall survival than those with low expression (*P = 0.05*) (**Figure 3F**).

To experimentally validate the interaction between miR-1246 and PPP2CB, we performed western blotting analysis. The results demonstrated that overexpression of miR-1246 in HLECs significantly reduced PPP2CB protein levels, while knockdown of miR-1246 significantly increased them compared to their respective controls (**Figure 3G**). To further confirm this finding, we performed a bioinformatic analysis which predicted a binding site for miR-1246 within the 3’-UTR of PPP2CB mRNA (**Figure 3H**). We then constructed luciferase reporter plasmids containing either the wild-type (PPP2CB-3’-UTR_wt_) or a mutated (PPP2CB-3’-UTR_mut_) version of this putative binding site. Luciferase reporter assays in HEK293T cells showed that co-transfection with a miR-1246 mimic significantly suppressed the luciferase activity of the PPP2CB-3’-UTR_wt_ reporter (*P < 0.01*), but had no effect on the activity of the PPP2CB-3’-UTR_mut_ reporter (*P = 0.996*) (**Figure 3I**). In conclusion, these results provide compelling evidence that miR-1246 specifically binds to the 3’-UTR of PPP2CB mRNA and directly suppresses its expression.

### 3.4 PPP2CB suppresses the biological behavior of lymphatic endothelial cells *in vitro*

We next investigated the functional role of PPP2CB in HLECs *in vitro*. Initially, qRT-PCR analysis confirmed that all three tested siRNA sequences effectively knocked down PPP2CB expression in HLECs, with si-2 and si-3 demonstrating the most significant knockdown efficiency; these two were therefore selected for subsequent experiments (**Figure 4A**). Functional assays, including scratch wound healing, transwell migration, matrigel invasion, and lymphatic tube formation, revealed that knockdown of PPP2CB significantly enhanced the migratory (**Figures 4C, D**), invasive (**Figure 4E**), and lymphangiogenic (**Figure 4F**) capacities of HLECs (*P < 0.05*). Conversely, overexpression of PPP2CB (**Figure 4B**) led to a significant suppression of HLECs migration (**Figures 4G, H**), invasion (**Figure 4I**), and tube formation (**Figure 4J**) (*P < 0.05*).

**Figure 4:**
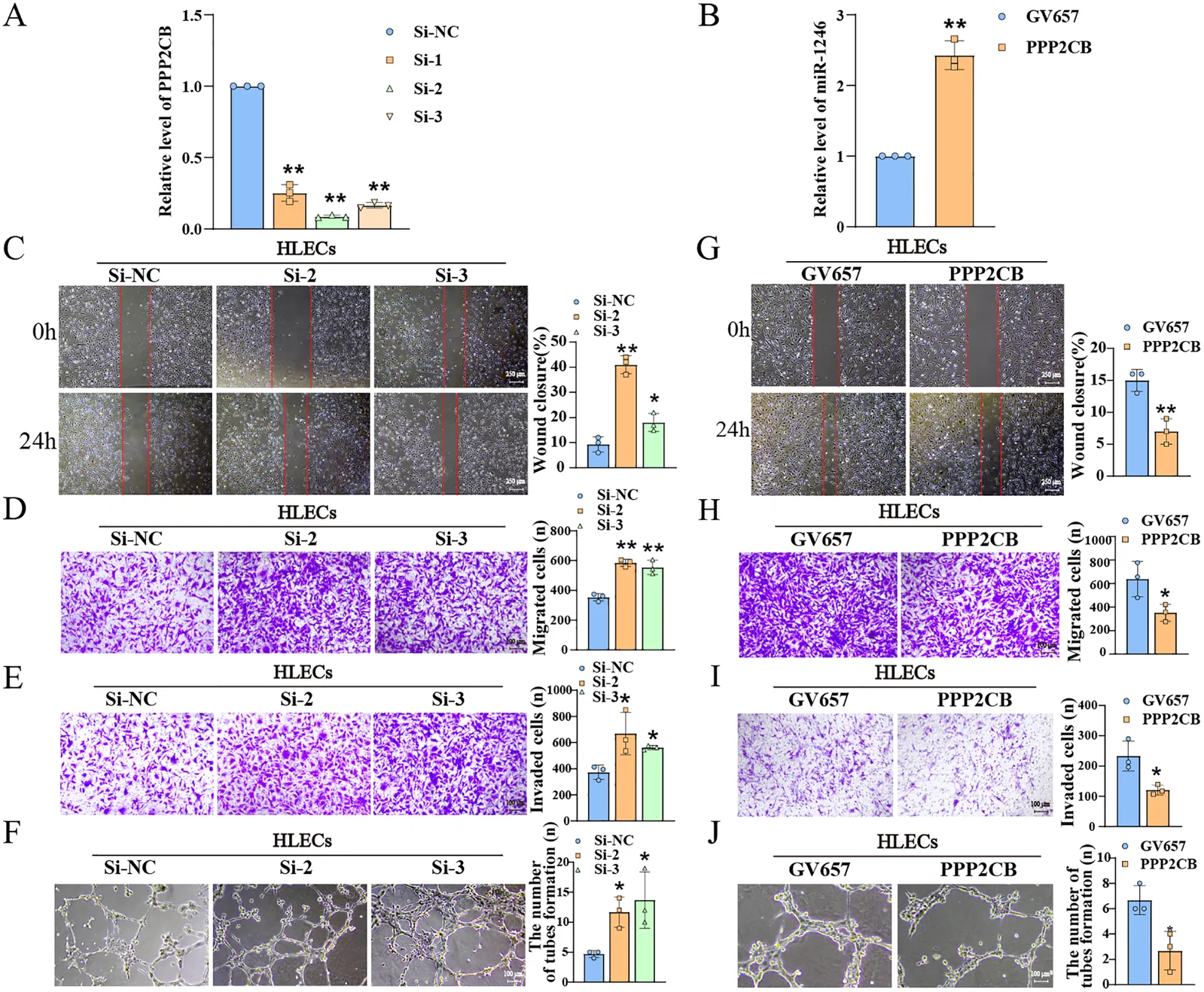
Effect of PPP2CB on migration, invasion, and lymphatic vessel formation of HLECs *in vitro*. **(A)** PPP2CB expression in HLECs transfected with si-NC, si-PPP2CB-1, si-PPP2CB-2, and si-PPP2CB-3 was detected by qRT-PCR. **(B)** PPP2CB expression in HLECs transfected with control group (GV657) and PPP2CB overexpression plasmid was detected by qRT-PCR. **(C–J)** The effects of PPP2CB on migration, invasion, and lymphatic vessel formation of HLECs detected by wound healing **(C, G)**, transwell **(D, H)**, Matrigel invasion **(E, I)**, and lymphatic vessel formation assays **(F, J)**. *P < 0.05 and **P < 0.01 versus the control groups.

### 3.5 Extracellular vesicle-derived miR-1246 from esophageal squamous cell carcinoma affects lymphatic endothelial cell behavior by targeting PPP2CB

We next performed rescue experiments to further validate that EV-derived miR-1246 exerts its effects by regulating PPP2CB in HLECs. The results showed that co-culture of HLECs with EVs derived from miR-1246-knockdown KYSE-150 cells (KYSE-150-_anti-miR-1246_-EVs) significantly attenuated HLECs migration, invasion, and lymphangiogenesis compared to the control group (KYSE-150-_anti-vector_-EVs). However, this inhibitory effect was significantly reversed when HLECs were concurrently transfected with PPP2CB-targeting siRNA (si-PPP2CB) (**Figure 5A**). Conversely, when we co-cultured HLECs with EVs derived from miR-1246-overexpressing KYSE-150 cells (KYSE-150-_LV-miR-1246_-EVs), the migratory, invasive, and lymphangiogenic capacities of the cells were significantly increased compared to those in the control group (KYSE-150-_LV-vector_-EVs). However, the concurrent transfection of a PPP2CB-overexpressing plasmid during the co-culture of KYSE-150-_LV-miR-1246_-EVs with HLECs significantly reversed the promotive effects of miR-1246 overexpression on the migratory, invasive, and lymphangiogenic capacities of HLECs (**Figure 5B**). These results indicate that miR-1246 derived from EVs of esophageal squamous cell carcinoma cells affects the biological behavior of lymphatic endothelial cells by targeting PPP2CB.

**Figure 5:**
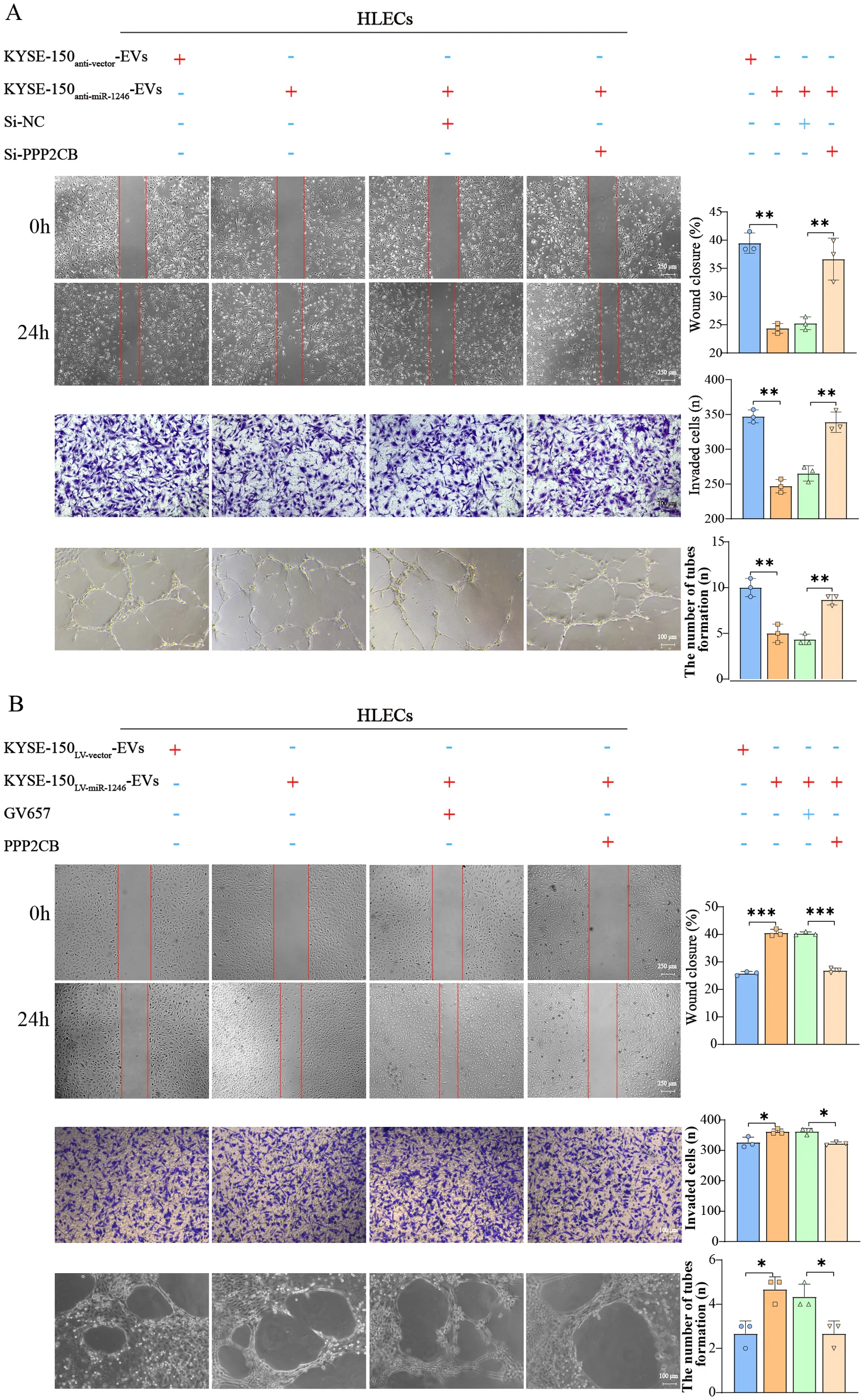
Effects of PPP2CB reversing miR-1246 derived from EVs on HLECs.**(A,B)** The effects of EVs-derived miR-1246 on migration, invasion, and lymphatic vessel formation of HLECs were reversed by the transfection of PPP2CB-targeting siRNA (si-PPP2CB) **(A)** and PPP2CB-overexpressing plasmid **(B)**.

### 3.6 EV-derived miR-1246 promotes lymph node metastasis of esophageal squamous cell carcinoma *in vivo* by targeting PPP2CB

The mouse footpad is an ideal site for modeling LNM due to its dense network of lymphatic vessels (19), which unidirectionally drain to popliteal and iliac lymph nodes, enabling the study of metastatic progression. To investigate the role of EV-derived miR-1246 in ESCC patients with LNM *in vivo*, we established a popliteal LNM model in nude mice (**Figure 6A**). KYSE-150 cells were inoculated into the footpad, and after palpable tumor formation, mice were randomly divided into four groups. These groups received intratumoral injections of KYSE-150-_LV-vector_-EVs, KYSE-150-_LV-miR-1246_-EVs, KYSE-150-_anti-vector_-EVs, and KYSE-150-_anti-miR-1246_-EVs. The results showed that the popliteal lymph nodes in the KYSE-150-_anti-miR-1246_-EVs group were significantly smaller and lighter than those in the KYSE-150-_anti-vector_-EVs control group. Conversely, lymph nodes in the KYSE-150-_LV-miR-1246_-EVs group were significantly larger and heavier than those in the KYSE-150-_LV-vector_-EVs control group (*P < 0.05*; **Figures 6B–D**).

**Figure 6:**
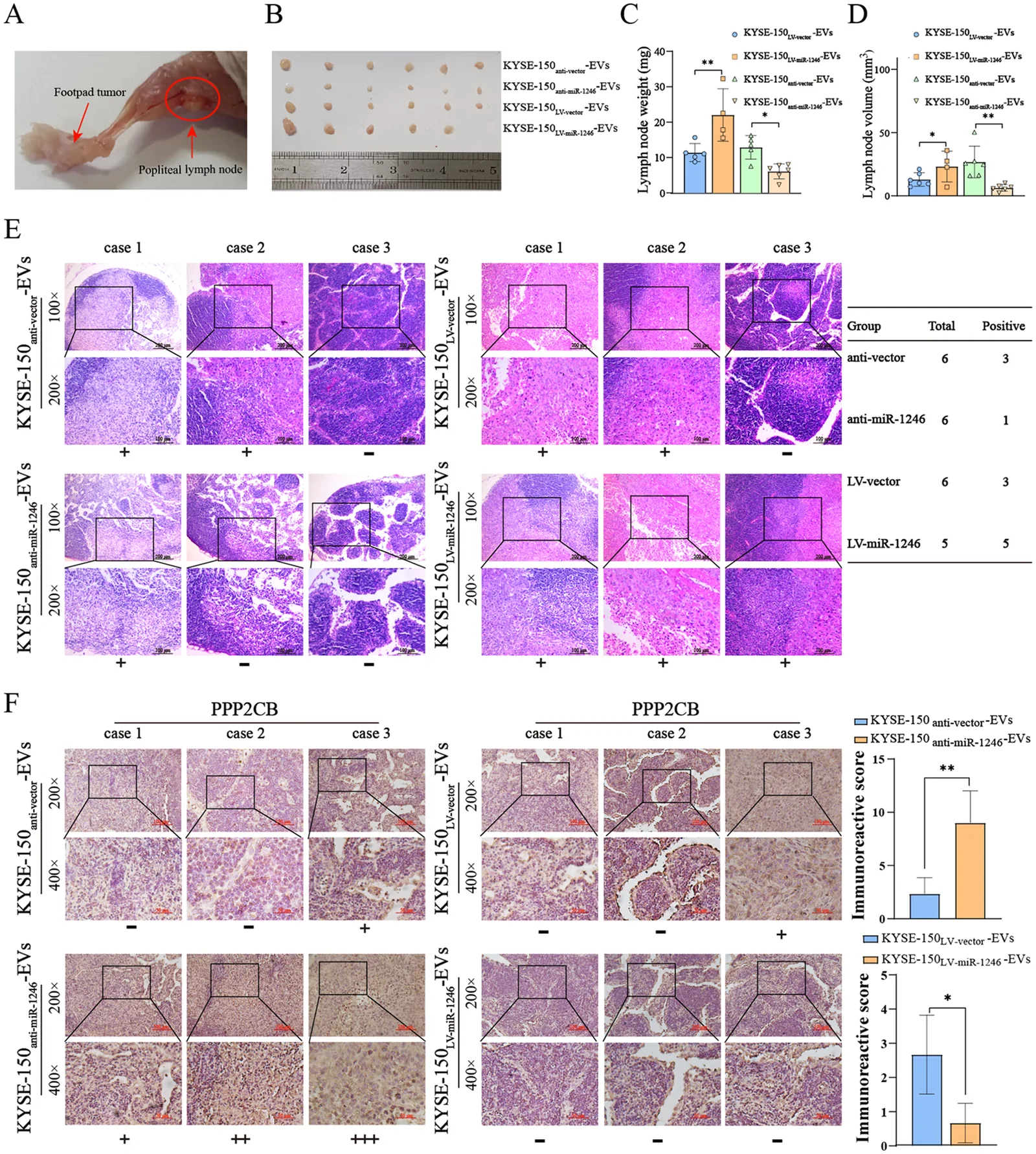
Effect of miR-1246 derived from EVs on lymph node metastasis of ESCC. **(A)** Schematic diagram of the mouse popliteal lymph node metastasis model. **(B)** Popliteal lymph node in each group of mice. **(C, D)** Weight **(C)** and volume **(D)** of metastatic tumors in popliteal lymph nodes in each group of mice. **(E)** Histopathological detection of metastatic foci in popliteal lymph nodes by hematoxylin-eosin (HE) staining. **(F)** The expression of PPP2CB proteins (brown) in tumor tissues of lymph node from of nude mice. n = 5 for the LV-miR-1246-EVs group, and n = 6 for all other experimental groups.

To rule out interference from inflammatory reactive hyperplasia of lymph nodes, all popliteal lymph-node specimens were subjected to H&E staining. Metastatic tumor foci within lymph nodes were identified and counted by a pathologist (**Figure 6E**). Histological observations showed a trend of increased metastatic foci in the KYSE-150-_LV-miR-1246_-EVs group and a trend of decreased foci in the KYSE-150-_anti-miR-1246_-EVs group. These findings suggest that EV-derived miR-1246 may potentially promote metastatic colonization of tumor cells in popliteal lymph nodes.

To further explore the relationship between PPP2CB and LNM, we assessed PPP2CB expression in the metastatic lymph nodes using immunohistochemistry. PPP2CB staining was observed in both the nucleus and cytoplasm of tumor cells. The number of PPP2CB-positive tumor cells was significantly lower in the KYSE-150-_LV-miR-1246_-EVs group compared to the KYSE-150-_LV-vector_-EVs group, whereas it was significantly higher in the KYSE-150-_anti-miR-1246_-EVs group than in the KYSE-150-_anti-vector_-EVs control group (**Figure 6F**). In conclusion, these results further demonstrate that EV-derived miR-1246 affects LNM of ESCC *in vivo* by targeting PPP2CB.

### 3.7 Investigation of the signaling pathways involving PPP2CB

In the present study, we initially explored the effects of miR-1246 and PPP2CB on the lymph node metastasis (LNM)-associated biological behaviors of ESCC *in vitro* and *in vivo*, and verified their association with ESCC patients with LNM. Subsequently, we further elucidated the underlying molecular mechanisms governing this process.

To identify the signaling pathways enriched by differentially expressed genes (DEGs) upon PPP2CB overexpression, next-generation sequencing was performed in HLECs with stable PPP2CB overexpression (**Figure 7A**). Sequencing results revealed a high degree of consistency in the global gene expression profiles between the two groups. The majority of genes (gray dots) exhibited no statistically significant differences in expression between the groups, whereas a limited subset of genes was identified as significantly differentially expressed (**Figure 7B**). Specifically, 89 genes were significantly downregulated and 106 genes were significantly upregulated (**Figure 7C**). These DEGs were further subjected to GO and KEGG pathway enrichment analyses using the DAVID database, with visualization conducted via Cytoscape software. The most significantly enriched terms in the BP, CC, and MF categories, along with their corresponding enrichment scores, are illustrated in **Figure 7D**. These results suggest that genes regulated by PPP2CB are involved in key biological functions, including “Golgi-associated vesicle” and “transforming growth factor-β receptor signaling pathway”. KEGG pathway enrichment analysis indicated that PPP2CB may regulate the biological processes of ESCC patients with LNM through multiple cancer-related signaling pathways, including glycerophospholipid metabolism, the NF-κB signaling pathway, and the Hippo signaling pathway (**Figure 7E**).

**Figure 7:**
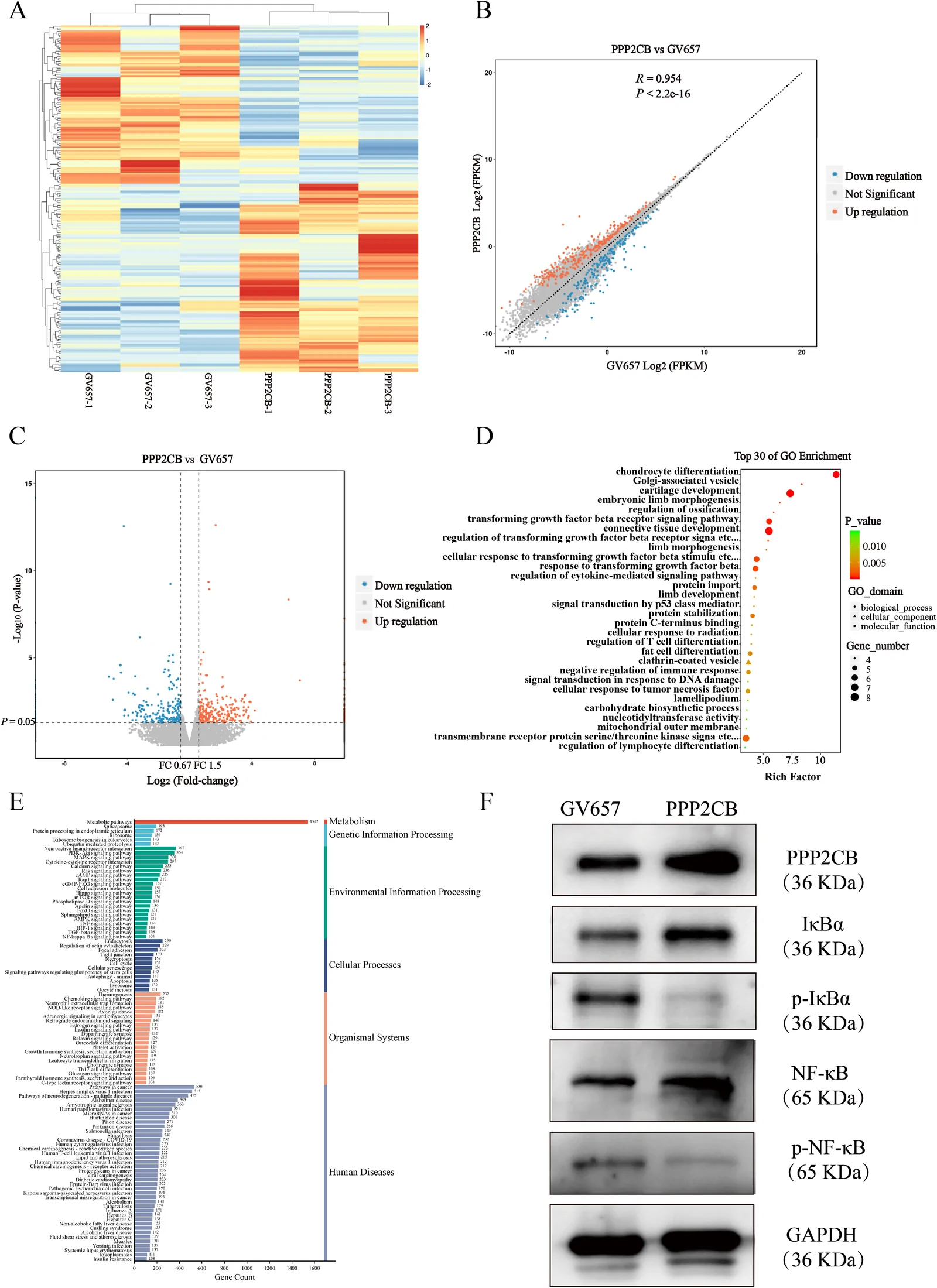
The potential mechanism of PPP2CB affects lymph node metastasis of ESCC. **(A)** Sequencing profiles of the PPP2CB-overexpressing group and the control group in HLECs. **(B)** Scatter plot showing global gene expression correlation between PPP2CB-overexpressing and control HLECs; gray dots: no significant expression difference; red/blue dots: significantly differentially expressed genes. **(C)** Statistics of differentially expressed genes (DEGs) in PPP2CB-overexpressing HLECs *vs*. control: 89 downregulated and 106 upregulated genes. **(D)** Top 30 Gene Ontology (GO) terms of biological processes (BP), cellular components (CC), and molecular functions (MF) for PPP2CB-related DEGs. **(E)** The pathway analysis results of PPP2CB-related DEGs. **(F)** The protein level of IκBα and NF-κB in PPP2CB-overexpressing HLEC cells.

Furthermore, previous studies have demonstrated that inhibition of PP2A activity leads to the upregulation of NF-κB-mediated transcriptional activity (20). To further characterize the mechanism by which PPP2CB regulates NF-κB signaling, we performed western blotting analysis for key components of the IκBα/NF-κB cascade. The results showed that PPP2CB overexpression significantly increased the protein level of total IκBα while reducing the phosphorylation level of IκBα in HLECs. Consequently, overexpression of PPP2CB also led to a marked decrease in phosphorylated NF-κB, without substantial alteration in total NF-κB expression (**Figure 7F**). These results indicate that PPP2CB may suppress NF-κB pathway activation by inhibiting IκBα phosphorylation and thereby reducing its degradation.

In conclusion, PPP2CB exerts a crucial regulatory role in ESCC patients with LNM and may modulate several tumor-associated signaling pathways, with the NF-κB pathway as one of its key downstream effectors.

### 3.8 Investigation of the miR-1246-PPP2CB-NF-κB regulatory axis

To establish the complete upstream-downstream relationship among miR-1246, PPP2CB and NF-κB signaling, we next performed molecular rescue experiments. The PPP2CB overexpression plasmid (GV657-PPP2CB) used in this assay contains only the PPP2CB coding sequence (CDS) without the endogenous 3’-UTR. Transcription is terminated by vector-derived poly-A signal, therefore, exogenous PPP2CB mRNA escapes targeting and repression by miR-1246. Western-blot results showed that miR-1246 overexpression induced NF-κB activation, manifested as elevated p-IκBα and p-NF-κB levels. Enforced expression of PPP2CB alone repressed NF-κB activity. Importantly, PPP2CB overexpression could partially reverse miR-1246-triggered excessive activation of NF-κB signaling (**Figure 8A**). These rescue findings confirm that miR-1246 activates the NF-κB cascade by down-regulating PPP2CB.

**Figure 8:**
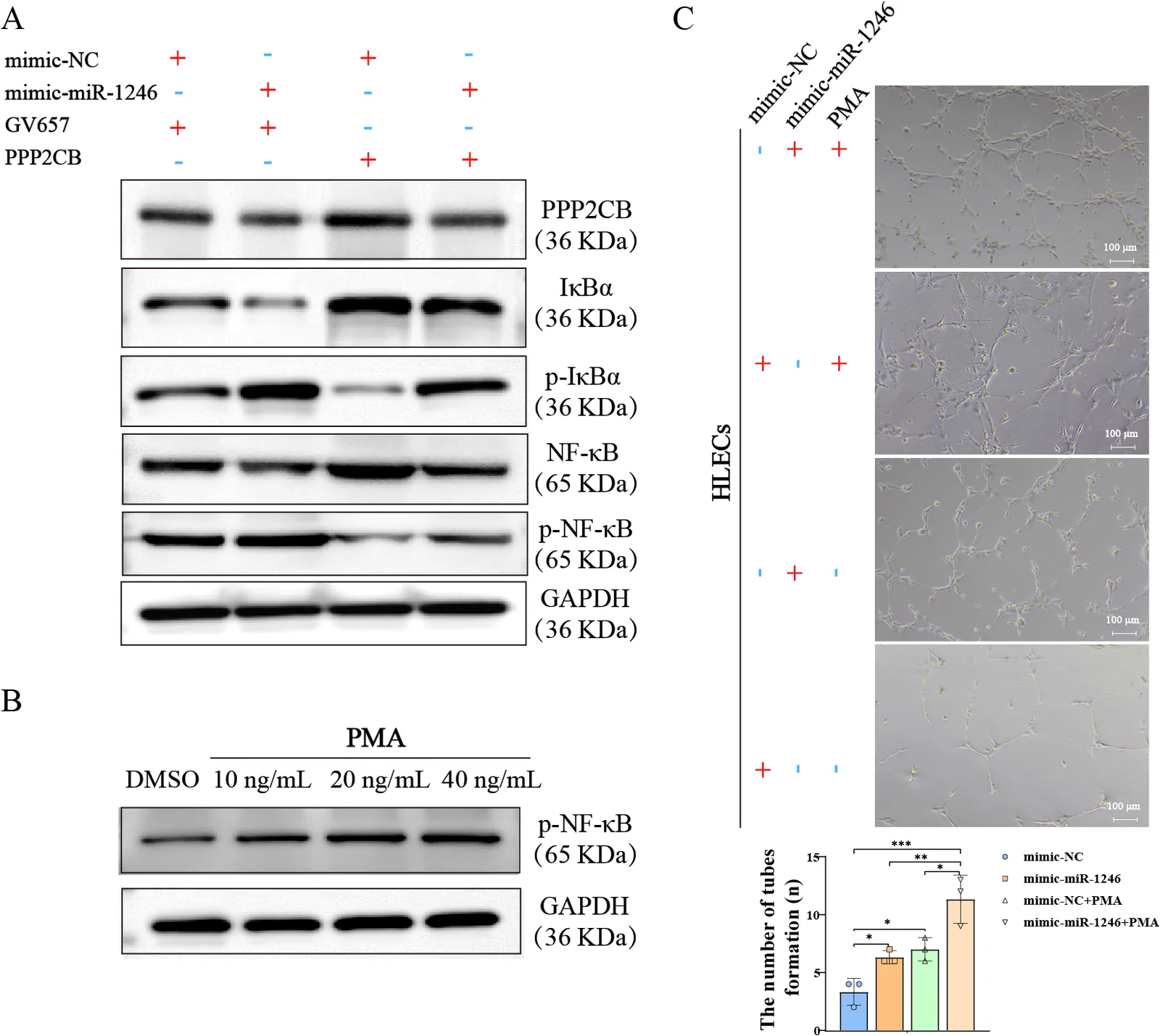
Verification of the miR-1246-PPP2CB-NF-κB signaling cascade regulating lymphangiogenesis. **(A)** The alterations in phosphorylation and total protein levels of NF-κB signaling components in HLECs were detected after miR-1246 overexpression and/or PPP2CB overexpression. **(B)** Western blot assay was used to screen the optimal concentration of PMA via detecting p-NF-κB expression. **(C)** The tube-formation capacity of HLECs was evaluated following miR-1246 overexpression combined with PMA treatment.

To verify whether NF-κB acts as a downstream functional effector mediating miR-1246-promoted lymphangiogenesis, we performed a tube-formation rescue assay using PMA, an agonist of the NF-κB signaling pathway. We set a concentration gradient of 10, 20, and 40 ng/mL for screening and selected the optimal drug concentration (20 ng/mL) (**Figure 8B**) that could stably upregulate the protein level of p-NF-κB. Subsequent tube-formation results showed that miR-1246 overexpression enhanced the tube-forming capacity of lymphatic endothelial cells; combined treatment with NF-κB agonist further potentiated the pro-lymphangiogenic phenotype induced by miR-1246 (**Figure 8C**). These results indicate that NF-κB activation is an important downstream effector involved in the pro-lymphangiogenic function of miR-1246.

## 4 Discussion

In recent years, the role of EVs and their cargo in LNM has emerged as a forefront area in cancer research (21–24). EVs carry bioactive molecules, including proteins, lipids, and nucleic acids, playing a crucial role in intercellular communication. Within the tumor microenvironment, tumor-derived EVs can be directly taken up by LECs. By transferring effector molecules such as miRNAs and proteins, these EVs activate LEC proliferation and migration, thereby promoting lymphangiogenesis (25–30).

Numerous studies have demonstrated that tumor cells secrete EVs that play a pivotal role in lymphangiogenesis and LNM (23, 31). Research has revealed that exosome-derived miR-363-5p inhibits cell proliferation and migration by targeting platelet-derived growth factor B (PDGFB) to regulate PDGF signaling activity, thereby exerting a suppressive effect on LNM of breast cancer (32). Beyond miRNAs, proteins carried by EVs are also instrumental. For instance, EVs secreted by melanoma cells are enriched in MET protein, which promotes LNM by activating the ERK pathways (33). Another study has shown that miR-99a encapsulated in EVs derived from colorectal cancer cells can target NLRP2 in fibroblasts, activate the NF-κB signaling pathway, thereby inducing the formation of an inflammatory microenvironment and promoting lymph node metastasis (34). These findings collectively underscore EVs and their bioactive cargo as critical regulators of LNM, providing a rationale for developing therapeutic strategies targeting EV biogenesis, secretion, or uptake.

Our study systematically reveals the critical role of EV-derived miR-1246 in ESCC lymph node metastasis. Through cohort validation, we identified a specific elevation of miR-1246 in plasma EVs from ESCC patients with LNM. Functional experiments demonstrated that ESCC cell-derived EVs deliver miR-1246 to HLEC cells, significantly enhancing their migratory, invasive, and tube-forming capabilities. Animal studies further confirmed that inhibiting miR-1246 markedly reduces ESCC lymph node metastasis. Mechanistically, we identified PPP2CB as a direct target of miR-1246. PP2A is a key tumor suppressor that maintains cellular homeostasis by regulating signaling pathways such as NF-κB. Previous research indicated that the loss of PP2A enzyme activity promotes tumor invasion and metastasis (35, 36). We have validated the miR-1246-PPP2CB-NF-κB regulatory axis at both molecular and cellular phenotypic levels. Our findings demonstrate that EV-derived miR-1246 targets and represses PPP2CB expression to trigger NF-κB signaling activation, which facilitates lymphatic endothelial cell activation and lymphangiogenesis, thereby contributing to lymph node metastasis in ESCC.

Considering the heterogeneous cellular origin of circulating plasma EVs, we further clarified the source of elevated EV-miR-1246 in ESCC patients. Firstly, our previous study verified that miR-1246 is specifically overexpressed in ESCC cells (6). Secondly, miR-1246 is a non-canonical miRNA generated from RNU2–1 degradation independently of classic miRNA biogenesis. It is selectively packaged into EVs by tumor cells, while immune, and stromal cells rarely produce or secrete this molecule (37). Thirdly, EV-miR-1246 was specifically elevated only in patients with lymph node metastasis, exhibiting a tumor-dependent expression pattern that excludes non-specific secretion from stromal or immune cells. Collectively, these results indicate that circulating EV-miR-1246 mainly originates from ESCC tumor cells.

From the perspective of clinical translation, the findings of this study hold dual significance: plasma EV-derived miR-1246, as a stable and easily accessible liquid biopsy biomarker, provides a novel approach for the early noninvasive diagnosis and prognostic evaluation of lymph node metastasis in ESCC. Meanwhile, EV-derived miR-1246 and its target gene PPP2CB may serve as potential therapeutic targets, offering experimental evidence for the development of novel intervention strategies.

Naturally, this study also has certain limitations. First, the specific downstream effector molecules and signaling pathways regulated by the miR-1246-PPP2CB axis, including the detailed regulatory mechanism of the NF-κB signaling pathway, still need further clarification. Second, the PKH67 lipophilic dye used for EV uptake imaging has inherent technical limitations: it may theoretically form dye aggregates or label non-EV lipid particles, and fluorescence imaging alone cannot definitively distinguish intact EV internalization from dye transfer (38). To address these technical caveats, we further performed qRT-PCR to quantify intracellular miR-1246 expression and examined EV-triggered functional alterations in HLECs. These complementary assays supported our observation that HLECs are capable of EV uptake. Third, although our functional rescue experiments support miR-1246 as a major functional cargo in ESCC cell-derived EVs, genetic manipulation of miR-1246 in donor cells may simultaneously alter EV secretion, protein composition, or other RNA components; therefore, the potential synergistic contribution of non-miR-1246 cargo cannot be completely excluded. Fourth, the clinical translational value of plasma EV-derived miR-1246 needs to be verified in prospective, large-sample clinical cohorts.

## 5 Conclusion

In summary, this study reveals a novel molecular mechanism by which esophageal squamous cell carcinoma-derived EVs contribute to lymph node metastasis by delivering miR-1246. By targeting and repressing PPP2CB, miR-1246 activates the NF-κB signaling pathway to drive lymphatic endothelial activation and lymphangiogenesis, which in turn facilitates lymph node metastasis in ESCC. This finding not only enriches our understanding of the molecular mechanisms underlying tumor lymphatic metastasis but also provides potential biomarkers and intervention targets for the diagnosis and treatment of esophageal squamous cell carcinoma with lymph node metastasis.

## Data Availability

The dataset has been submitted to the Science Data Bank (ScienceDB). The DOI is: https://doi.org/10.57760/sciencedb.012dg.

## References

[1] Bray F Laversanne M Sung H Ferlay J Siegel RL Soerjomataram I . Global cancer statistics 2022: GLOBOCAN estimates of incidence and mortality worldwide for 36 cancers in 185 countries. CA Cancer J Clin. (2024) 74:229–63. doi: 10.3322/caac.21834 38572751

[2] Zhang Y Liu L Han M Li L Wu Q Wang X . Unraveling the power of radiomics: prediction and exploration of lymph node metastasis in stage T1/2 esophageal squamous cell carcinoma. NPJ Precis Oncol. (2025) 9:176. doi: 10.1038/s41698-025-00929-2 40523899 PMC12170826

[3] Teng H Xue M Liang J Wang X Wang L Wei W . Inter- and intratumor DNA methylation heterogeneity associated with lymph node metastasis and prognosis of esophageal squamous cell carcinoma. Theranostics. (2020) 10:3035–48. doi: 10.7150/thno.42559 32194853 PMC7053185

[4] Greening DW Xu R Rai A Suwakulsiri W Chen M Simpson RJ . Clinical relevance of extracellular vesicles in cancer - therapeutic and diagnostic potential. Nat Rev Clin Oncol. (2025) 22:924–52. doi: 10.1038/s41571-025-01074-2 41062719

[5] De Giorgis V Barberis E Manfredi M . Extracellular vesicles proteins for early cancer diagnosis: From omics to biomarkers. Semin Cancer Biol. (2024) 104-105:18–31. doi: 10.1016/j.semcancer.2024.07.003 39074601

[6] Wang Y Li X Wei X Li L Bai H Yan X . Identification of combinatorial miRNA panels derived from extracellular vesicles as biomarkers for esophageal squamous cell carcinoma. MedComm. (2023) 4:e377. doi: 10.1002/mco2.377 37731947 PMC10507283

[7] Takeshita N Hoshino I Mori M Akutsu Y Hanari N Yoneyama Y . Correction to: Serum microRNA expression profile: miR-1246 as a novel diagnostic and prognostic biomarker for oesophageal squamous cell carcinoma. Br J Cancer. (2024) 131:1106. doi: 10.1038/s41416-024-02820-6 39187688 PMC11405385

[8] Kauko O Westermarck J . Non-genomic mechanisms of protein phosphatase 2A (PP2A) regulation in cancer. Int J Biochem Cell Biol. (2018) 96:157–64. doi: 10.1016/j.biocel.2018.01.005 29355757

[9] Sarais F Krempien F Koehn C Brewing L Classen CF Walter M . Targeting PP2A in cancer: an underrated option. J Exp Clin Cancer Res. (2025) 44:295. doi: 10.1186/s13046-025-03560-y 41146310 PMC12557939

[10] Khan A Zhang Y Ma N Shi J Hou Y . NF-κB role on tumor proliferation, migration, invasion and immune escape. Cancer Gene Ther. (2024) 31:1599–610. doi: 10.1038/s41417-024-00811-6 39033218

[11] Su C Chen Z Luo H Su Y Liu W Cai L . Different patterns of NF-κB and Notch1 signaling contribute to tumor-induced lymphangiogenesis of esophageal squamous cell carcinoma. J Exp Clin Cancer Res. (2011) 30:85. doi: 10.1186/1756-9966-30-85 21939555 PMC3215933

[12] Yee YH Chong SJF Kong LR Goh BC Pervaiz S . Sustained IKKβ phosphorylation and NF-κB activation by superoxide-induced peroxynitrite-mediated nitrotyrosine modification of B56γ3 and PP2A inactivation. Redox Biol. (2021) 41:101834. doi: 10.1016/j.redox.2020.101834 33838472 PMC8056462

[13] Wynne K Mooney L Fitzsimons S Halasz M Aluri ER Brougham DF . A comparative analysis of extracellular vesicles (EVs) from human and feline plasma. Sci Rep. (2022) 12:10851. doi: 10.1038/s41598-022-14211-z 35761023 PMC9237114

[14] Ten A Kumeiko V Farniev V Gao H Shevtsov M . Tumor microenvironment modulation by cancer-derived extracellular vesicles. Cells. (2024) 13:682. doi: 10.3390/cells13080682 38667297 PMC11049026

[15] Lipinski S Tiemann K . Extracellular vesicles and their role in the spatial and temporal expansion of tumor-immune interactions. Int J Mol Sci. (2021) 22:3374. doi: 10.3390/ijms22073374 33806053 PMC8036938

[16] Wang SE . Extracellular vesicles in cancer therapy. Semin Cancer Biol. (2022) 86:296–309. doi: 10.1016/j.semcancer.2022.06.001 35688334 PMC10431950

[17] Seshacharyulu P Pandey P Datta K Batra SK . Phosphatase: PP2A structural importance, regulation and its aberrant expression in cancer. Cancer Lett. (2013) 335:9–18. doi: 10.1016/j.canlet.2013.02.036 23454242 PMC3665613

[18] Watt LF Panicker N Mannan A Copeland B Kahl RGS Dun MD . Functional importance of PP2A regulatory subunit loss in breast cancer. Breast Cancer Res Treat. (2017) 166:117–31. doi: 10.1007/s10549-017-4403-5 28744751

[19] Lin Y Xue J Liao S . Blocking lymph flow by suturing afferent lymphatic vessels in mice. J Vis Exp. (2020) 23:e61178. doi: 10.3791/61178 32478754

[20] Xu C Tang J Yang Q Zhao H Liu Y Cao J . Histone deacetylase 5 deacetylates the phosphatase PP2A for positively regulating NF-κB signaling. J Biol Chem. (2021) 297:101380. doi: 10.1016/j.jbc.2021.101380 34740611 PMC8634046

[21] Kalluri R LeBleu VS . The biology, function, and biomedical applications of exosomes. Science. (2020) 367:aau6977. doi: 10.1126/science.aau6977 32029601 PMC7717626

[22] Perez Hurtado EC Henao Agudelo JS Foganholi da Silva RA Viração TA Fernandes C . The role of extracellular vesicles in cancer. Curr Top Membr. (2024) 94:247–85. doi: 10.1016/bs.ctm.2024.06.010 39370209

[23] Cao LM Qiu YZ Li ZZ Wang GR Xiao Y Luo HY . Extracellular vesicles: Hermes between cancers and lymph nodes. Cancer Lett. (2025) 623:217735. doi: 10.1016/j.canlet.2025.217735 40268131

[24] Wang L Li L Zhu G . Role of extracellular vesicles on cancer lymphangiogenesis and lymph node metastasis. Front Oncol. (2021) 11:721785. doi: 10.3389/fonc.2021.721785 34552874 PMC8451414

[25] Primorac D Brlek P Bulić L Hrvatin N Škaro V Projić P . Clinical applications of extracellular vesicles: Promises and pitfalls. Int J Mol Sci. (2026) 27:1509. doi: 10.3390/ijms27031509 41683929 PMC12897622

[26] García-Silva S Benito-Martín A Nogués L Hernández-Barranco A Mazariegos MS Santos V . Melanoma-derived small extracellular vesicles induce lymphangiogenesis and metastasis through an NGFR-dependent mechanism. Nat Cancer. (2021) 2:1387–405. doi: 10.1038/s43018-021-00272-y 34957415 PMC8697753

[27] Xiao Z Feng X Zhou Y Li P Luo J Zhang W . Exosomal miR-10527-5p inhibits migration, invasion, lymphangiogenesis and lymphatic metastasis by affecting Wnt/β-catenin signaling via Rab10 in esophageal squamous cell carcinoma. Int J Nanomedicine. (2023) 18:95–114. doi: 10.2147/IJN.S391173 36636641 PMC9831078

[28] Lodha P Acari A Rieck J Hofmann S Dieterich LC . The lymphatic vascular system in extracellular vesicle-mediated tumor progression. Cancers (Basel). (2024) 16:4309. doi: 10.3390/cancers16234039 39682225 PMC11640020

[29] Yao W Jia X Zhu L Xu L Zhang Q Xia T . Exosomal circ_0026611 contributes to lymphangiogenesis by reducing PROX1 acetylation and ubiquitination in human lymphatic endothelial cells (HLECs). Cell Mol Biol Lett. (2023) 28:13. doi: 10.1186/s11658-022-00410-z 36803975 PMC9936748

[30] Wang CA Tsai SJ . Regulation of lymphangiogenesis by extracellular vesicles in cancer metastasis. Exp Biol Med. (2021) 246:2048–56. doi: 10.1177/15353702211021022 34139872 PMC8524766

[31] Su X Brassard A Bartolomucci A Dhoparee-Doomah I Qiu Q Tsering T . Tumour extracellular vesicles induce neutrophil extracellular traps to promote lymph node metastasis. J Extracell Vesicles. (2023) 12:e12341. doi: 10.1002/jev2.12341 37563798 PMC10415595

[32] Wang X Qian T Bao S Zhao H Chen H Xing Z . Circulating exosomal miR-363-5p inhibits lymph node metastasis by downregulating PDGFB and serves as a potential noninvasive biomarker for breast cancer. Mol Oncol. (2021) 15:2466–79. doi: 10.1002/1878-0261.13029 34058065 PMC8410538

[33] Peinado H Alečković M Lavotshkin S Matei I Costa-Silva B Moreno-Bueno G . Melanoma exosomes educate bone marrow progenitor cells toward a pro-metastatic phenotype through MET. Nat Med. (2012) 18:883–91. doi: 10.1038/nm.2753 22635005 PMC3645291

[34] Zhou M Liu H Hui J Chen Q Zhao Y Wang H . Extracellular vesicle-packaged miR-99a reprograms fibroblasts to create an inflammatory niche that drives colorectal cancer metastasis. Cancer Res. (2026) 86:131–45. doi: 10.1158/0008-5472.CAN-25-0663 40991395

[35] Wu X Huang S Gao J Huang S Chen L Zhao Z . AKR1B10 dictates c-Myc stability to suppress colorectal cancer metastasis via PP2A nitration. Sci Adv. (2025) 11:eadv6937. doi: 10.1126/sciadv.adv6937 40845116 PMC12372902

[36] Zhang Y Wang X Li A Guan Y Shen P Ni Y . PP2A regulates metastasis and vasculogenic mimicry formation via PI3K/AKT/ZEB1 axis in non-small cell lung cancers. J Pharmacol Sci. (2022) 150:56–66. doi: 10.1016/j.jphs.2022.07.001 36055752

[37] Xu YF Hannafon BN Khatri U Gin A Ding WQ . The origin of exosomal miR-1246 in human cancer cells. RNA Biol. (2019) 16:770–84. doi: 10.1080/15476286.2019.1585738 30806147 PMC6546371

[38] Shimomura T Seino R Umezaki K Shimoda A Ezoe T Ishiyama M . New lipophilic fluorescent dyes for labeling extracellular vesicles: Characterization and monitoring of cellular uptake. Bioconjug Chem. (2021) 32:680–4. doi: 10.1021/acs.bioconjchem.1c00068 33719402

